# Functional Study of Mammalian Neph Proteins in *Drosophila melanogaster*

**DOI:** 10.1371/journal.pone.0040300

**Published:** 2012-07-06

**Authors:** Martin Helmstädter, Kevin Lüthy, Markus Gödel, Matias Simons, Ashish, Deepak Nihalani, Stefan A. Rensing, Karl-Friedrich Fischbach, Tobias B. Huber

**Affiliations:** 1 Renal Division, University Hospital Freiburg, Freiburg, Germany; 2 Faculty of Biology, University of Freiburg, Freiburg, Germany; 3 BIOSS Centre for Biological Signalling Studies, Albert-Ludwigs-University Freiburg, Freiburg, Germany; 4 Center for Systems Biology, University of Freiburg, Freiburg, Germany; 5 CSIR-Institute of Microbial Technology, Chandigarh, India; 6 Renal Electrolyte and Hypertension Devision, University of Pennsylvania, Philadelphia, Pennsylvania, United States of America; Lancaster University, United Kingdom

## Abstract

Neph molecules are highly conserved immunoglobulin superfamily proteins (IgSF) which are essential for multiple morphogenetic processes, including glomerular development in mammals and neuronal as well as nephrocyte development in *D. melanogaster*. While *D. melanogaster* expresses two Neph-like proteins (Kirre and IrreC/Rst), three Neph proteins (Neph1–3) are expressed in the mammalian system. However, although these molecules are highly abundant, their molecular functions are still poorly understood. Here we report on a fly system in which we overexpress and replace endogenous Neph homologs with mammalian Neph1–3 proteins to identify functional Neph protein networks required for neuronal and nephrocyte development. Misexpression of Neph1, but neither Neph2 nor Neph3, phenocopies the overexpression of endogenous Neph molecules suggesting a functional diversity of mammalian Neph family proteins. Moreover, structure-function analysis identified a conserved and specific Neph1 protein motif that appears to be required for the functional replacement of Kirre. Hereby, we establish *D. melanogaster* as a genetic system to specifically model molecular Neph1 functions *in vivo* and identify a conserved amino acid motif linking Neph1 to *Drosophila* Kirre function.

## Introduction

**Figure 1:**
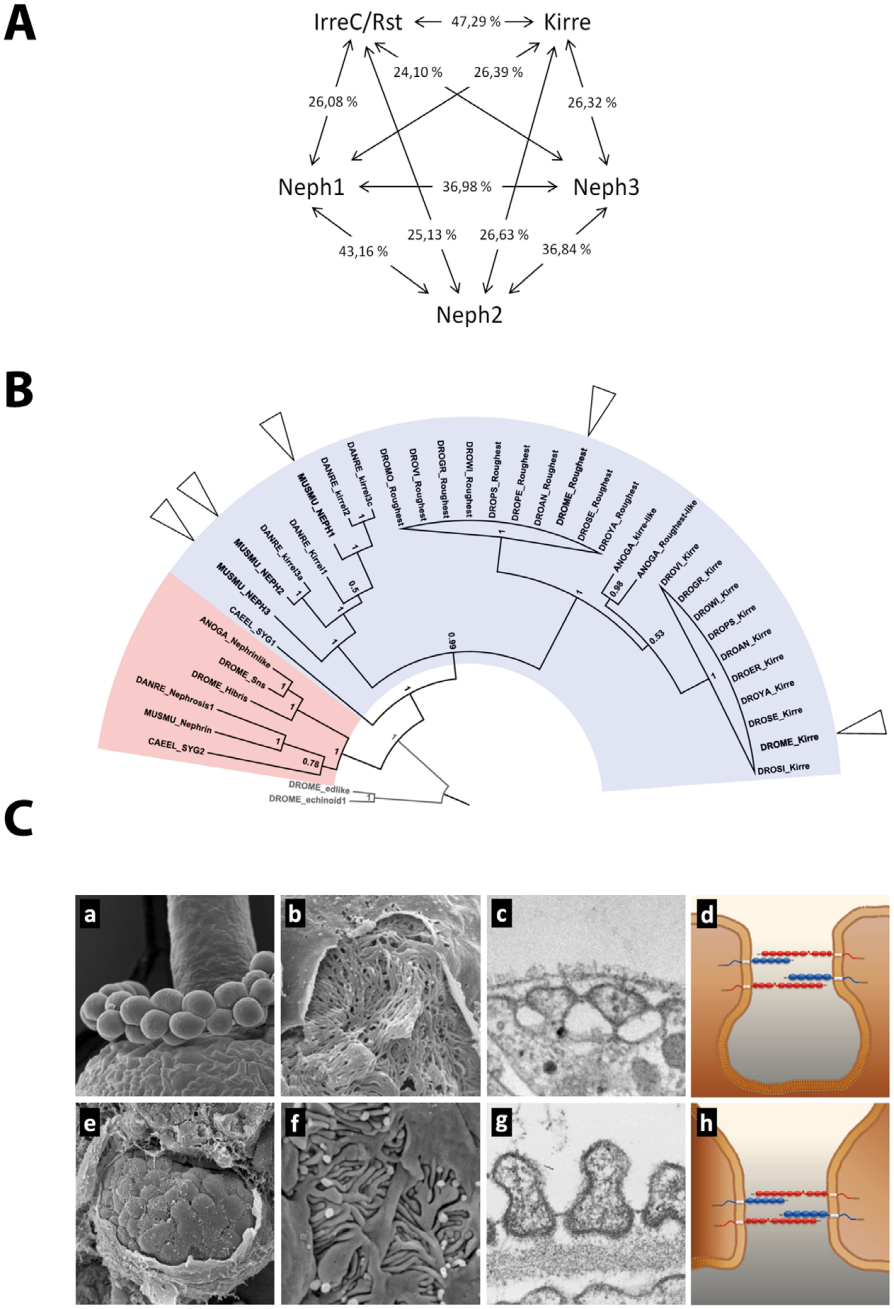
The irre cell recognition module (IRM) is conserved across species. **A.** Similarity network of Neph1–3, Kirre and IrreC/Rst generated with T-Coffee multiple sequence alignment algorithm. Values represent full length protein similarity in percent. **B.** Protein tree of the Neph (blue) and Nephrin (red) protein families as present in *Mus musculus*, *Danio rerio*, *Caenorhabditis elegans, Anopheles gambiae* and several *Drosophila* species *(Drosophila mojavensis* (DROMO), *Drosophila virilis* (DROVI), *Drosophila grimshawi* (DROGR), *Drosophila willistoni* (DROWI), *Drosophila pseudoobscura* (DROPS), *Drosophila persimilis* (DROPE), *Drosophila ananassae* (DROAN), *Drosophila melanogaster* (DROME), *Drosophila sechellia* (DROSE), *Drosophila yakuba* (DROYA). Arrowheads mark the proteins investigated in this study. **C.** Structural comparison of *M. musculus* kidney glomerulus (**e**), podocytes (**f**) and slit diaphragm (**g**) with GCNs of *D. melanogaster* (**a**). The rough surface of the GCN underneath the basement membrane (**b**) is formed by the nephrocyte diaphragm (**c**). In contrast to the mammalian slit diaphragm which is formed between neighboring podocytes (**h**), the *Drosophila* nephrocyte diaphragm is formed within one GCN (**d**).

The IgSF proteins of Nephrin and Neph families (also called IRM proteins [1]) have been conserved throughout Metazoan evolution. All Nephrin and Neph proteins share extracellular immunoglobulin-like domains and a short cytoplasmic tail that contains multiple signaling motifs [2], [3]. The extracellular domains of Nephrin and Neph proteins bind to each other in *cis*- and/or *trans*- interactions [4]. Two Neph homologs (IrreC/Rst, Kirre) and two Nephrin homologs (Hbs, Sns) are involved in pupal eye development, muscle fusion and axonal guidance in *Drosophila* [5]–[9]. In *C. elegans,* synapse development and synaptic target recognition also employ members of the Nephrin-Neph protein family. In this system the Nephrin homolog SYG-2 and the Neph1 homolog SYG-1 mediate precise recognition of appropriate partners and trigger synapse formation of the hermaphrodite specific motor neuron (HSNL) [10], [11]. Interestingly, all mammalian Neph molecules (Neph1–3) have been shown to be able to functionally replace endogenous SYG-1 indicating a potential redundant function of Neph proteins [12], [13]. Despite the diversity of signaling mechanisms and expression patterns of IRM proteins throughout different species, some important themes are beginning to emerge: a striking property of IRM proteins is the formation of variable, homo- and heterophilic interaction modules in *cis* and *trans* conformation that precisely guide cellular connections [4], [12]–[15]. An interesting example of such a highly specialized cell-cell contact is the slit diaphragm at the kidney filtration barrier, which consists of *cis* and *trans-* interacting Nephrin and Neph1 molecules (Fig. 1C e-h). Mutations in Nephrin lead to congenital nephrotic syndrome which is characterized by a disruption of the kidney filtration barrier, kidney failure and severe protein loss into the urine [16]. In addition, mice lacking Neph1 are proteinuric and reveal effacement of podocyte foot processes [17]. Nephrin and Neph1 molecules have been demonstrated to form a *cis-* and *trans-* interacting complex [4], [18], [19]. Moreover, the Nephrin-Neph1 protein complex has been linked to several signaling processes at the slit diaphragm, like actin regulation, polarity signaling and cell survival [20], [21]. Strikingly, recent investigations revealed that Sns and Kirre form a filtration slit in Garland cell nephrocytes (GCNs) of *Drosophila* (Fig. 1C a-d) which is very similar to the mammalian slit diaphragm of podocytes [15], [22], [23]. As the experimental accessibility of the mammalian slit diaphragms is very limited, this finding has important implications. Therefore, the *Drosophila* GCN appears to be an ideal system to study Nephrin-Neph protein functions in a genetically easy tractable system [24]. Interestingly, Sns and Kirre are involved not only in the slit diaphragm formation of GCNs, but also mediate the fusion process of GCNs that results in binuclear GCNs [15].

We employed *D. melanogaste*r as a model system to investigate the evolutionary conservation of the Nephrin-Neph proteins and to determine differences between the mammalian Neph proteins 1–3 in their ability to phenocopy and rescue Kirre and IrreC/Rst phenotypes. Our results demonstrate that Neph1 is the only mammalian Neph protein that can mimic the phenotypes of overexpressed Kirre or misexpressed IrreC/Rst in GCNs. Furthermore, we illustrate that only misexpressed Neph1, which partially colocalizes with Sns in GCNs, is able to rescue a GCN fusion phenotype caused by the loss of Kirre. A bioinformatic search for cytoplasmic motifs present in Kirre, IrreC/Rst and Neph1 revealed a conserved 12 AA motif (KIN1 motif), which seems to be required for Kirre and Neph1 function.

## Results

### Overexpression of Kirre and Misexpression of IrreC/Rst, Neph1, but not Neph2 or Neph3, Leads to an Irregular Fusion of GCNs

A multiple sequence alignment of Neph1–3, Kirre and IrreC/Rst did not reveal a significantly higher sequence similarity for any of the Neph proteins in comparison to Kirre or IrreC/Rst protein (Fig. 1A). In order to investigate differences between the three Neph proteins, we created fly lines in which transcription of Neph1–3 proteins is under control of the UAS-sequence. We used the GAL4/UAS-System to misexpress V5-tagged versions of the mammalian Neph proteins in different test systems.

**Figure 2:**
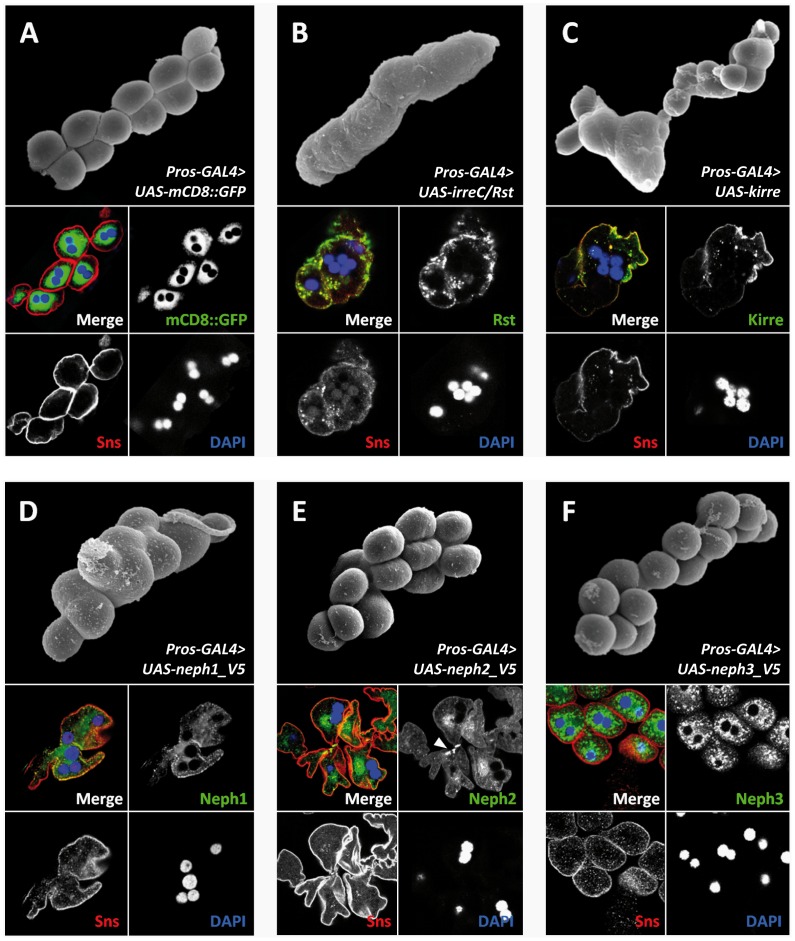
IRM protein misexpression in GCNs can induce clustering and/or hyperfusion. **A-F.** Scanning electron micrograph of *Drosophila* GCNs at third larval stage and immunoreactivity of the corresponding genotypes. The control shows the distribution of misexpressed membrane associated mCD8::GFP. The misexpressed protein is endocytosed if it is not stabilized in the nephrocyte diaphragm. The binucleate GCNs are separated (**A**). IrreC/Rst misexpression leads to clustering and hyperfusion of GCNs (**B**). Kirre overexpression leads to a similar phenotype. (**C**). Neph1 misexpression also leads to clustering and fusion of GCNs (**D**)**.** Neph2 misexpression does not interfere with the fusion of GCNs (**E**). The arrow head marks the enriched Neph2 immunoreactivity at cell-cell contacts. Misexpression of Neph3 does not interfere with the GCN fusion. Immunoreactivity shows that the Neph3 expression pattern is similar to the GFP control (**F**).

During embryonic development mononucleate GCNs fuse to form binucleate GCNs at the third larval stage (Fig. 2A). The fusion process is mediated by the heterophilic interaction of Kirre and Sns [15]. Overexpression of Kirre driven by *pros-GAL4* resulted in a gain of fusion phenotype at the level of 3^rd^ instar larvae (Fig. 2C). Similarly, the misexpression of IrreC/Rst protein, which normally is not expressed in GCNs [15], resulted in enhanced cell fusion (Fig. 2B). However, out of the three mammalian Neph proteins, only Neph1 proved to induce the fusion phenotype (Fig. 2D). To exclude dose dependent differences the transcript levels of ectopically expressed Neph proteins were quantified by qRT-PCR using primers specific to the V5 tag. This quantitation revealed comparable expression levels of the three Neph proteins with a slightly higher amount of Neph3 mRNA (Fig. S1). The control staining of membrane associated mCD8::GFP illustrates that transmembrane proteins which are not stabilized within the nephrocyte diaphragm are mainly enriched in vesicles due to the high endocytosis rate of GCNs (Fig. 2A). Interestingly, Neph1 colocalized with Sns, suggesting a stabilization in the nephrocyte diaphragm by heterophilic interaction with Sns. Neph2 was partially enriched at cell-cell-contacts without causing enhanced fusion (Fig. 2E); misexpressed Neph3 neither colocalized with Sns, nor did it interfere with the fusion process (Fig. 2F).

### Neph1 Can Rescue the GCN *kirre^-^* Phenotype

**Figure 3:**
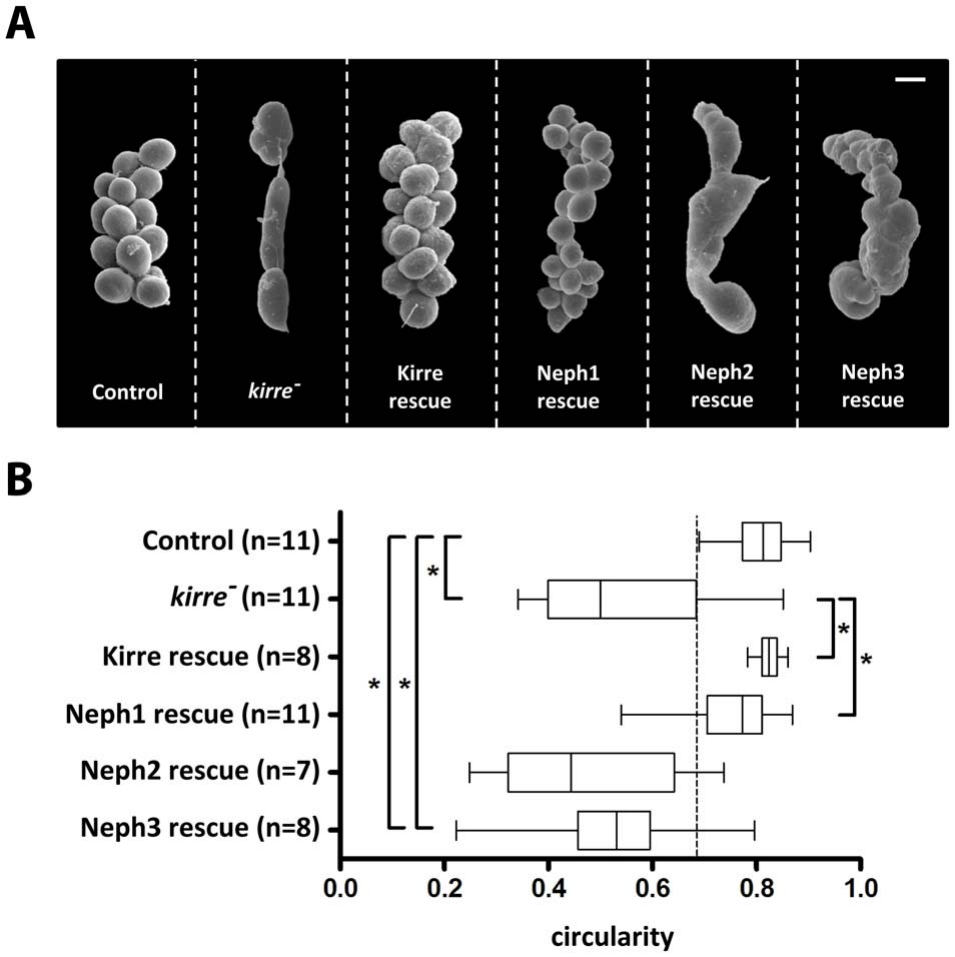
Third larval stage *kirre^-^* GCNs are hyperfused. Neph1 can rescue the *kirre^-^* phenotype. **A.** Scanning electron micrographs of GCNs at third larval stage. Scale bar: 20 µm. **B.** The fact that hyperfused GCNs lose their spherical shape was used to quantify the rescue efficiency. Kirre or Neph1 expression is sufficient to significantly rescue the *kirre^-^* phenotype. *P value <0,0001 (unpaired t-test with Welchs correction). Genotypes: Control: *+/sns-GAL4. kirre^-^*: *Df(1)duf* *^sps-1^/y*. Kirre rescue: *Df(1)duf* *^sps-1^/y; UAS-Kirre/sns-GAL4.* Neph1 rescue: *Df(1)duf* *^sps-1^/y; UAS-neph1/sns-GAL4*. Neph2 rescue: *Df(1)duf* *^sps-1^/y; UAS-neph2/sns-GAL4*. Neph3 rescue: *Df(1)duf* *^sps-1^/y; UAS-neph3/sns-GAL4.*

GCNs express Kirre but do not express IrreC/Rst. Lack of Kirre in *kirre^-^* flies leads to a severe hyperfusion phenotype in the third larval stage that seems to be caused by irregularities during the Kirre-dependent fusion of mononucleate GCNs (Fig. 3A). To further characterize the evolutionary conservation of Neph1 and Kirre at the functional level we used the hyperfusion phenotype for rescue experiments. We found that Neph1 expression using the GCN driver *sns-GAL4* is sufficient to rescue the *kirre^-^* phenotype (Fig. 3). In contrast to this successful rescue experiment, Neph2 or Neph3 expression is insufficient to restore correct GCN fusion. The hyperfused GCNs lose the typically spherical shape and this finding was used to quantify the rescue efficiency (Fig. 3B). These results further support the functional redundancy of Neph1 and Kirre.

### GCN Fusion Requires a Conserved Motif in the Cytoplasmic Tail of Neph Proteins

**Figure 4:**
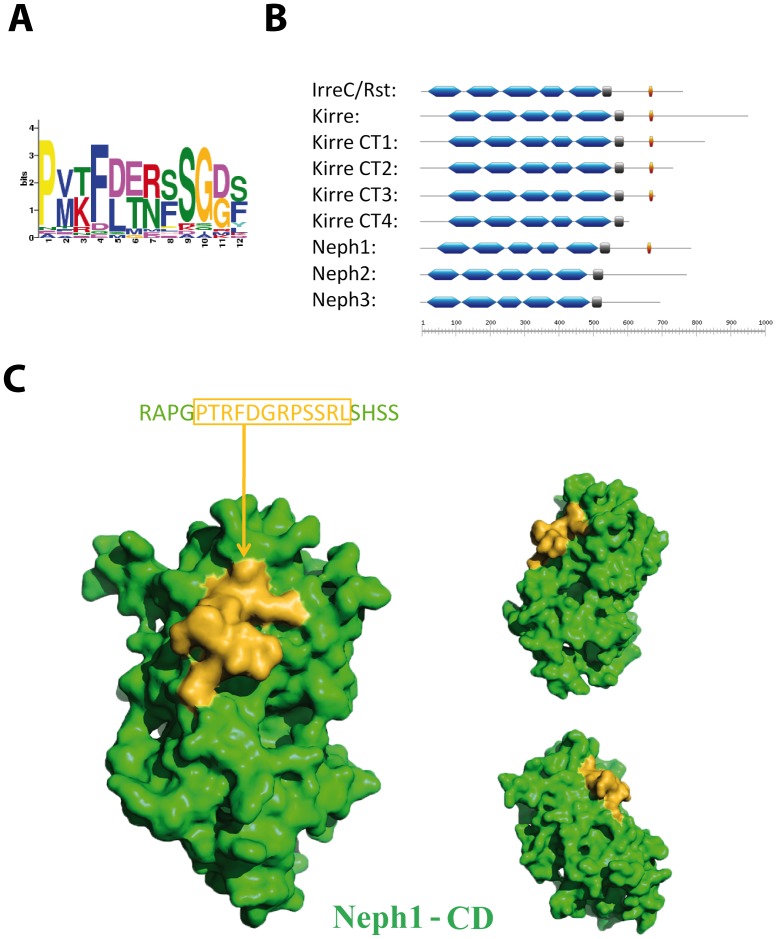
Schematic drawing of the identified KIN1 motif and its position in the protein sequences. **A.** MEME generated Bitlogo of the KIN1 motif. **B.** Structural comparison of Neph and Neph-like proteins. Blue: Ig domain. Grey: transmembrane domain. Yellow: KIN1 motif. Scale: number of amino acids. **C.** Surface representation of the structural model of the cytoplasmic domain of Neph1 is shown in green with the KIN1 motif highlighted in yellow.

**Figure 5:**
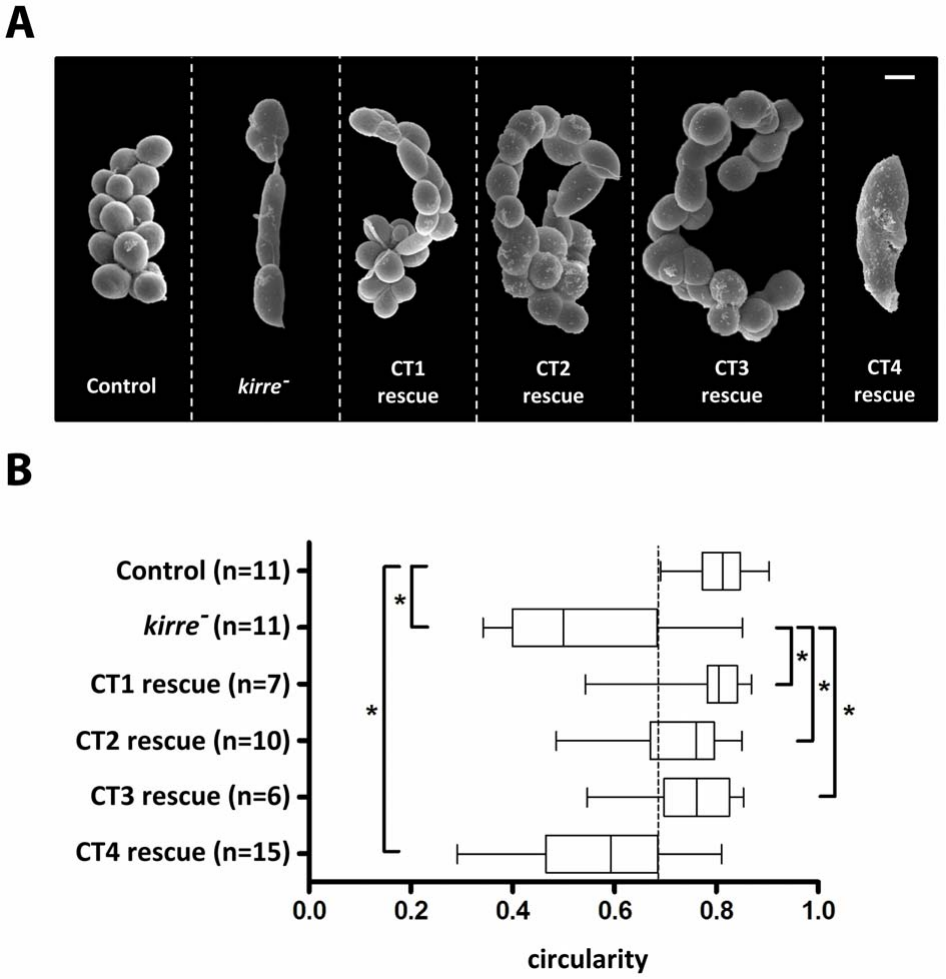
GCN fusion requires the cytoplasmic part of Kirre containing the KIN1 motif. **A.** Scanning electron micrographs of GCNs at third larval stage. Scale bar: 20 µm. **B.** Quantitation of GCN circularity revealing that Kirre versions CT1– CT3 which still contain the KIN1 motif are able to partially restore the wildtype situation. However, Kirre-CT4 missing the conserved KIN1 motif is not able to rescue the GCN fusion phenotype. *P value <0,0001 (unpaired t-test with Welchs correction). Genotypes: Control: *+/sns-GAL4*; *kirre^-^*: *Df(1)duf* *^sps-1^*; CT1 rescue: *Df(1)duf* *^sps-1^/y; UAS-CT1/sns-GAL4*; CT2 rescue: *Df(1)duf* *^sps-1^/y; UAS-CT2/sns-GAL4*; CT3 rescue: *Df(1)duf* *^sps-1^/y; UAS-CT3/sns-GAL4,* CT4 rescue: *Df(1)duf* *^sps-1^/y; UAS-CT4/sns-GAL4.*

An *in silico* search for motifs conserved between IrreC/Rst and Kirre of all *Drosophila* species and Neph1, but not present in Neph2 and Neph3, identified a 12 amino acid sequence which we named KIN1 motif for the proteins in which it occurs (Kirre: PVKFDERFSGDF; IrreC/Rst: PMTFLTNSSGGS; Neph1: PTRFDGRPSSRL) (Fig. 4). To determine the location of this motif in Neph1 we mapped its presence in the recently described structural model of the cytoplasmic domain of Neph1 [25]. Interestingly, this motif was localized in the exposed region of the molecule (Fig. 4C). This further suggests that it is readily accessible to potential interacting proteins and may have a functional role. To validate the importance of the KIN1 motif for the rescue ability of the entire protein, we used cytoplasmatically truncated Kirre versions to rescue the GCN phenotype of larva deficient in Kirre. The Kirre versions CT1– CT3 [26], which still contain the KIN1 motif (Fig. 4B), were able to partially restore the wildtype situation. However, Kirre-CT4 lacks the conserved KIN1 motif and was not able to rescue the GCN fusion phenotype, providing evidence for the functional importance of this cytoplasmic Neph1/Kirre motif (Fig. 5).

### Neph1 Misexpression can Mimic Neuronal and Eye Phenotypes of Overexpressed Kirre or IrreC/Rst

**Figure 6:**
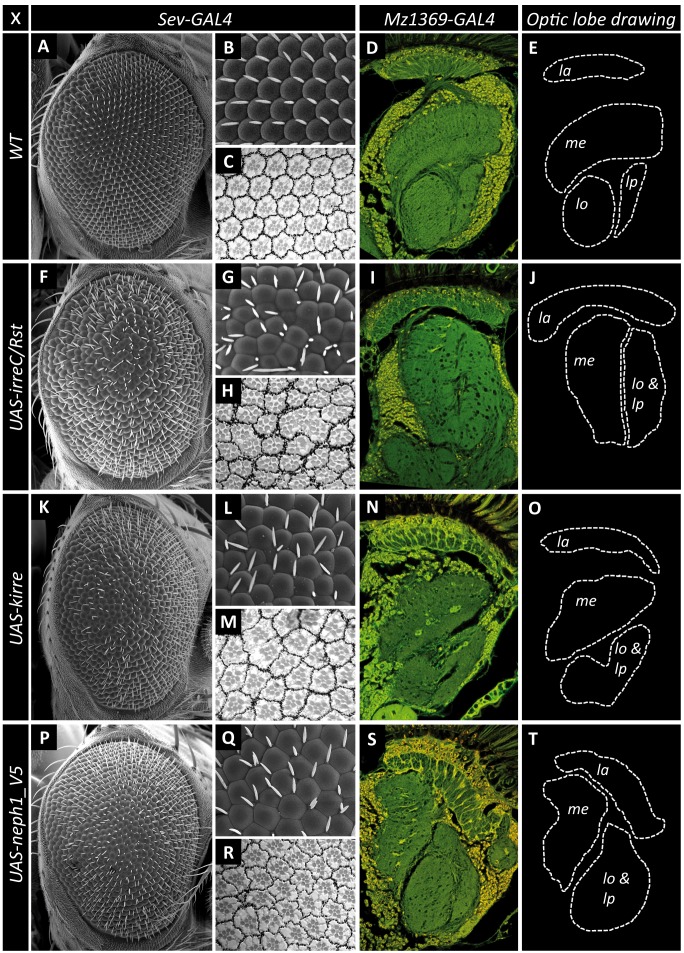
Neph1 can mimic neuronal and eye phenotypes of overexpressed Kirre or IrreC/Rst. Scanning electron micrograph of adult *Drosophila* eyes (**A,B,F,G,K,L,P,Q**), close-up of the eye (**B,G,L,Q**) and light micrographs of semithin sections (**C,H,M,R**). The control shows the regular crystal like arrangement of an *Drosophila* eye (**A,B**). *Sev-GAL4* induced misexpression of IrreC/Rst (**F,G**) or Kirre (**K,L**) results in a rough eye phenotype. Misexpression of Neph1 with *sev-GAL4* also causes a rough eye phenotype (**P,Q**). All three genotypes exhibit fusion of ommatidia (**H,M,R**). Genotypes: *sev-GAL4/+* (**A,B,C**); *sev-GAL4/UAS-irreC/rst* (**F,G,H**); *sev-GAL4/+,UAS-kirre/+* (**K,L**); *sev-GAL4/UAS-neph1_V5* (**P,Q,R**). Auto fluorescence micrographs of adult *Drosophila* optic lobes (**D,I,N,S**). The control fly shows the typical wildtype-like arrangement of the neuropils of the *Drosophila* optic lobe (**D,E**). Overexpression of Rst (**I**) or Kirre (**N**) causes severe misrouting of fibers in medulla and lobula complex and a disorganization of these neuropils (**J,O**). Misexpression of Neph1 leads to a similar phenotype (**S,T**). Optic lobe drawing: la: lamina, me: medulla, lo: lobula, lp: lobula plate. Genotypes: *Mz1369-GAL4/+* (**D**); *Mz1369-GAL4/UAS-rst* (**I**); *Mz1369-GAL4/+,UAS-kirre/+* (**N**); *Mz1369-GAL4/UAS-neph1_V5* (**S**).

To further investigate the functional redundancy of Neph1 and Kirre we utilized another established model system for Nephrin-Neph function, the *D. melanogaster* compound eye [8], [14], [27]. As shown previously [1], *sev-GAL4* induced misexpression of IrreC/Rst or Kirre results in a rough eye phenotype (Fig. 6F and K). Interestingly, misexpression of Neph1, similarly to the misexpression of IrreC/Rst or Kirre, also caused a rough eye phenotype (Fig. 6P). Semithin sections of rough eyes reveal fusion of ommatidia (Fig. 6H,M and R). To quantify the rough eye phenotype, clusters of bristles were counted. These data (Fig. S2) revealed that the severity of the rough eye phenotype in flies misexpressing Neph1 is comparable to flies misexpressing IrreC/Rst. To exclude position effects we tested four lines with independent insertions of the three Neph proteins. Using the driver line *Mz1369-GAL4* to drive transgene expression in neurons and glial cells, overexpression of IrreC/Rst resulted in a severe disorganization of all four neuropils of the optic lobe (Fig. 6I). Of these neuropils, the lamina seemed to be least affected, while in some cases the inner optic chiasm between medulla, lobula and lobula plate completely disappeared, leaving the impression of a fusion between these three otherwise clearly distinct neuropils (Fig. 6J). A similar phenotype of variable penetrance resulted from Kirre overexpression (Fig. 6N and O). Strikingly, Neph1 misexpression could phenocopy IrreC/Rst or Kirre overexpression (Fig. 6S and T), whereas Neph2 and Neph3 misexpression had no effect (Fig. S3).

## Discussion

The large spectrum of genetic and molecular tools that has evolved for the analysis of protein function in *D. melanogaster* is unique. As a complex multicellular organism with a sophisticated nervous system and kidney-like slit diaphragm harboring structures, *D.* *melanogaster* is an ideal system to study Nephrin-Neph protein function.

While in the *C. elegans* model all three mammalian Neph proteins are able to partially rescue the synaptic developmental defects of SYG-1 mutants [12], we demonstrate that in *D. melanogaster* only Neph1, and neither Neph2 nor Neph3, is able to mimic phenotypes produced by either IrreC/Rst or Kirre. This underscores the evolutionarily higher and more complex level of Neph protein function in *D. melanogaster* compared to *C. elegans*. Previously, our *in silico* data indicated that Neph proteins in vertebrates belong to three paralogous groups which probably evolved before or during the emergence of early vertebrates [12]. Such gene duplications often remove the selective pressure from copies, frequently resulting in diverging and non-redundant biological functions of the paralogs. Indeed, our data suggest that, although there are common functions of Neph molecules (as previously evidenced by the ability of all mammalian Neph isoforms to rescue the *SYG-1*-deficiency phenotype in *C. elegans*), different mammalian Neph proteins may fulfill rather diverse functions. However, future studies will have to delineate the precise functional diversity of Neph proteins in mammals.

Interestingly, the functional conservation between Neph1 and Kirre throughout metazoan evolution appears to be strong enough for Neph1 to rescue the *kirre^-^* GCN phenotype. From this finding we conclude that Neph1 is the functional ortholog of the *D.* *melanogaster* Neph-like Kirre. Like Kirre, Neph1 seems to interact with Sns and gets stabilized in the nephrocyte diaphragm. It seems to be a common feature of IRM proteins that overexpression phenotypes and knockdown/knockout phenotypes result in similar phenotypes [1]. In accordance with this, overexpression of Kirre and misexpression of IrreC/Rst and Neph1 in GCNs led to phenotypes similar to those resulting from loss of Kirre, indicating that the fusion process is highly dependent on the correct spatiotemporal expression level of IRM proteins. This also holds true for axonal pathfinding defects where loss and gain of function phenotypes phenocopy each other [9].

In summary, our results suggest that Neph1 shares functional features with *Drosophila* IrreC/Rst and Kirre which are absent in Neph2 and Neph3 proteins. By searching for motifs conserved in the three proteins Kirre, IrreC/Rst and Neph1, but missing in Neph2 and Neph3, we identified the 12AA KIN1 motif. The unique exposed position of the KIN1 motif at the surface in the three-dimensional structural model of Neph1 places it in an ideal position to interact with other proteins. Both the position of this motif within the sequence and the phylogeny of this gene family suggest that the common ancestor of insects and vertebrates possessed a gene harboring this motif. In the course of evolution the composition of the Neph protein family C-terminus was probably altered due to genetic insertions and deletions, leading e.g. to loss of the motif in Neph2/3. The fact that Kirre CT4 expression is insufficient to rescue the *kirre^-^* phenotype illustrates that the Kirre-dependent GCN fusion is not mediated by adhesion of the extracellular part only. Moreover, it points to an essential role of signaling via the cytoplasmic part for correct fusion, potentially via the KIN1 motif.

The easily tractable and functionally closely related podocyte-like GCN *Drosophila* system will be a powerful platform for applying further reverse genetic screens identifying regulators and functional domains important for Nephrin-Neph protein trafficking, localization, and signaling.

## Materials and Methods

### D. melanogaster Husbandry

*D. melanogaster* stocks were cultured on standard cornmeal molasses agar food and maintained at 25°C unless otherwise mentioned.

### Fly Stocks and Generation of Transgenic Flies

The V5-tagged mouse Neph1, 2 and 3 cDNAs were cloned to *pUAST* vectors [28] and injected into *w^-^* embryos by the company Genetivision to generate transgenic flies [29]. Insertions were mapped and homozygous strains were generated by repeated crosses with the balancer stock w[*]; Kr[If-1]/CyO; D [*1*] */TM6C,Sb* [*1*] *Tb* [*1*] *; (1;2;3)* (BSN7199). The resulting lines were subsequently crossed to *Mz1369-GAL4* (gift from Joachim Urban), *sev-GAL4* (gift from Konrad Basler), *prospero-GAL4* (gift from Barry Denholm) and *sns-GCN-GAL4* (gift from Susan Abmayr). The misexpression results were verified by the use of four different lines for each of the Neph proteins with each of these independent insertions causing a similar phenotype.

The *kirre^-^* strain *Df(1)duf* *^sps-1^* contains a small sequence deficiency removing the *duf* locus only (Prieto-Sànchez et al., in preparation) and was obtained by Mar Ruiz-Gómez via Barry Denholm. Kirre CT mutant flies were kindly shared by Sarada Bulchand.

### Immunostaining

Pupal eyes and GCNs (isolated from wandering third instar larvae) were dissected in PBS, fixed for 15 min in 4% paraformaldehyde, washed in PBST (PBS, 0,2%Triton X-100) and incubated with primary antibodies (in PBS, 0,2%Triton X-100, 0,05% sodium azide). A fluorescent-dye-coupled secondary antibody (Alexa Fluor 488, Alexa Fluor 568; Invitrogen) was used as the secondary antibody. Preparations were embedded in Vectashield (Vector Labs). The mounting medium for GCNs contained DAPI for nuclear staining. Samples were imaged using NIKON A1 CLEM with inverted microscope Eclipse TI. The following primary antibodies were used: Mouse anti-Roughest Mab24A5.1 (1∶10) [9]; rabbit anti-Kirre A126i (1∶200) [30]; mouse anti-SNS (1∶200) [31]; rabbit anti-SNS (1∶200) [31]; mouse anti-V5 (1∶200) (obtained from Invitrogen). The Rat anti-Hbs antibody (1∶500) was kindly shared by Tetsuya Tabata [32].

### Microscopic Examination and Quantitation

GCN preparations and adult eyes for SEM were fixed using Bouin solution. Dehydration with 70% EtOH, 80% EtOH, 90% EtOH and 100% EtOH, was followed by incubation in 50∶50 EtOH/HMDS. After incubation in 100% HMDS the solvent was allowed to evaporate. All samples were coated with gold using a Polaron Cool Sputter Coater E 5100. Samples were imaged using a Leo 1450 VP electron microscope. SEM data of GCNs were quantified by measuring the circularity of single nephrocytes after marking their cell shape with Adobe Photoshop. Sections of adult retinae were performed as previously described [33]. Eyes were sectioned through the equatorial region and imaged using NIKON A1 CLEM with inverted microscope Eclipse TI.

### Histology

For sectioning, adult flies were fixed, dehydrated, imbedded in paraffin and cut into 7 µm sections as described by Heisenberg and Böhl (1979). Sections were mounted on coated glass slides, and neuronal structures were visualized by auto fluorescence.

### Bioinformatics

Amino acid sequences were aligned using M.A.F.F.T. [34] and the alignment manually curated using Jalview [35]. The most appropriate model was evaluated using ProtTest [36] and turned out to be WAG+I+G+F. Tree reconstruction was carried out using Quicktree SD [37] with 1,000 bootstrap samples and with MrBayes [38] using WAG with eight gamma distributed rates and two hot and two cold chains stopping at an average standard deviation of split frequencies of 0.01. The Bayesian inference and neighbor-joining trees showed the same overall branching order with very minor differences. The Bayesian tree shown was visualized with FigTree (http://tree.bio.ed.ac.uk/software/figtree/). Motif detection was carried out using MEME [39] on the cytoplasmic part of the protein sequences, using *M. musculus* Neph2/3 as negative and *M. musculus* Neph1 as well as *Drosophila* Kirre and IrreC/Rst and *D.* *rerio* Kirre-like sequences as positive set. Using a manually curated alignment of *M. musculus* Neph, *D. rerio* Kirre-like and *D. melanogaster* Kirre and Roughest sequences, two motifs were confirmed to be present in Neph1 and Kirre/IrreC/Rst, but not in Neph2/3. The molecular modeling of the cytoplasmic domain of Neph1 was performed as recently described [25].

### RNA Isolation from 3^rd^ Instar Larvae and Quantitative RT-PCR

Whole 3^rd^ instar larvae were homogenized in Trizol using an Eppendorf homogenizer. RNA was purified with chloroform followed by DNase treatment. The design of V5-tag specific primers was carried out using the public software primer3 (Primer Selection Program http://fokker.wi.mit.edu/primer3/input.htm), cDNA was synthesized using a mixture of oligoT and random hexamer primers from 0,5 µg of total RNA using Superscript II (Invitrogen) following the manufacturer’s protocol. Real time PCR was performed for 45 cycles using Lightcycler 480 (Roche). The log2 ratios (transgene/wild-type fly) were calculated as described [40]. The actin gene was used for normalization. Individual PCR amplifications were carried out in duplicates and analyses included three biological replicas.

## Supporting Information

Figure S1 **Expression level quantitation of Neph1, Neph2 and Neph3.** The amount of Neph1, Neph2 and Neph3 mRNA was quantified by qRT-PCR. Results are presented as log2 ratio of eCp values obtained under transgene misexpression and control. Samples were normalized using the actin gene. The transcript levels are in a comparable range. The amount of Neph3 mRNA is slightly higher than Neph1 and Neph2 mRNA. Genotypes: *pros-GAL4/UAS-Neph1_V5*, *pros-GAL4/UAS-Neph2_V5* and *pros-GAL4/UAS-Neph3_V5*. N = 3.
(TIF)

Figure S2 **Rough eye phenotype quantitation**. To quantify the rough eye phenotype, clusters of bristles were counted. The severity of the rough eye phenotype in flies misexpressing Neph1 is comparable to that in flies overexpressing IrreC/Rst. For each Neph protein data of four independent insertions are shown. Arrows are marking the lines selected for the rescue experiments and qRT-PCR.
(TIF)

Figure S3 **Neph2 and Neph3 cannot mimic neuronal and eye phenotypes of overexpressed Kirre or IrreC/Rst.** Scanning electron micrographs of adult *Drosophila* eyes (**A,B,F,G**), close-up of the eye (**B,G**) and light micrographs of semithin sections (**C,H**). *sev-GAL4* induced misexpression of Neph2 does not cause a rough eye phenotype (**A,B**). *sev-GAL4* induced misexpression of Neph3 leads to a weak increase in the amount of misplaced bristles (**F,G,H**),(see Figure S2). Genotypes: *sev-GAL4/UAS-neph2_V5* (**A,B,C**), *sev-GAL4/UAS-neph3_V5* (**F,G,H**). Auto fluorescence micrographs of adult *Drosophila* optic lobes (**D,I**). The optic lobes show the typical wildtype-like arrangement of the neuropils. Optic lobe drawing: la: lamina, me: medulla, lo: lobula, lp: lobula plate. Genotypes: *Mz1369-GAL4/UAS-neph2_V5* (**D**), *Mz1369-GAL4/UAS-neph3_V5* (**I**).
(TIF)
