# Supplementary figures and images for: Functional Study of Mammalian Neph Proteins in Drosophila melanogaster
Source: PLoS One. 2012 Jul 6;7(7):e40300. doi: 10.1371/journal.pone.0040300 (PMC3391254; doi:10.1371/journal.pone.0040300)

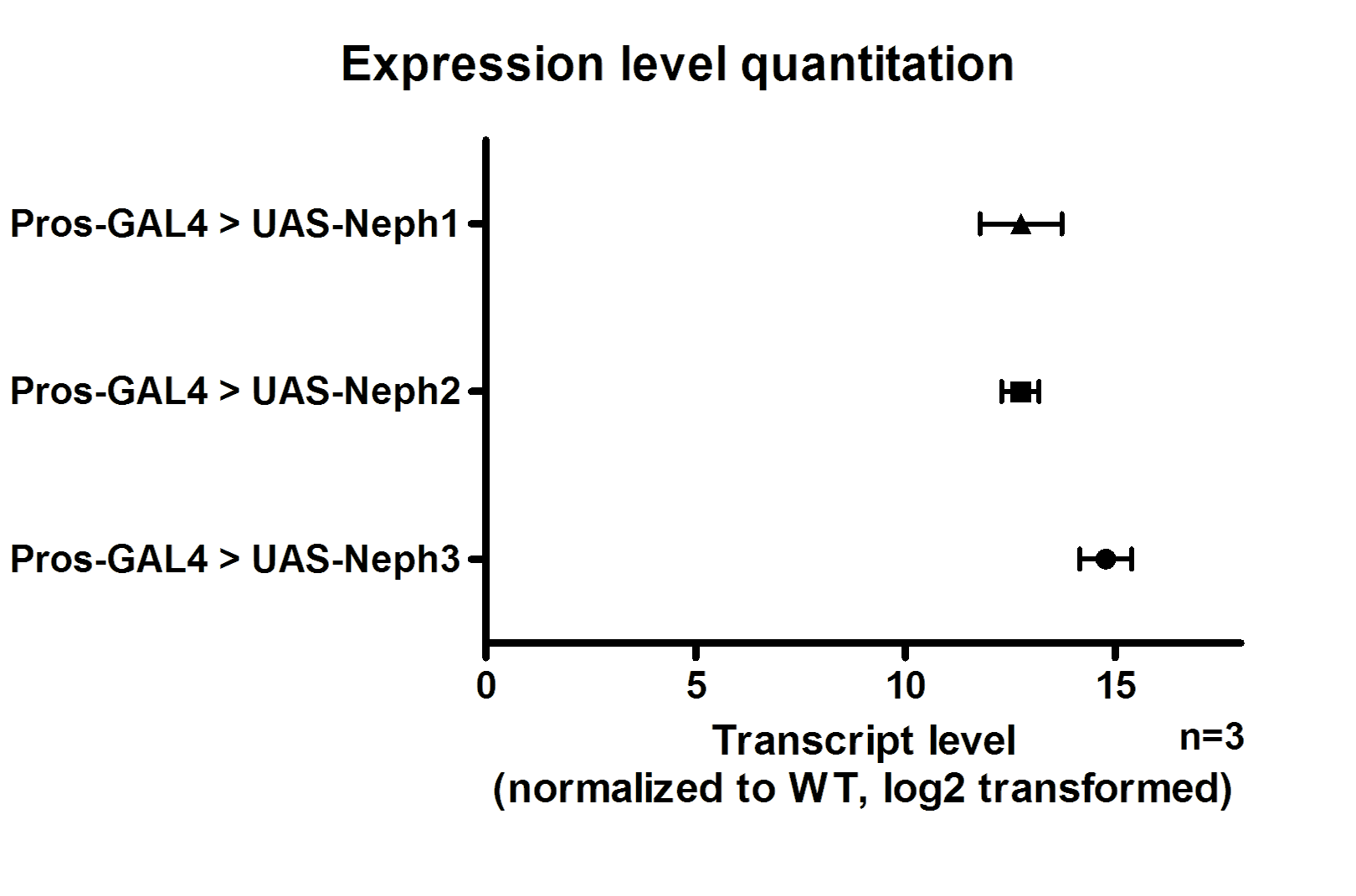

Supplement: Figure S1 — Expression level quantitation of Neph1, Neph2 and Neph3. The amount of Neph1, Neph2 and Neph3 mRNA was quantified by qRT-PCR. Results are presented as log2 ratio of eCp values obtained under transgene misexpression and control. Samples were normalized using the actin gene. The transcript levels are in a comparable range. The amount of Neph3 mRNA is slightly higher than Neph1 and Neph2 mRNA. Genotypes: pros-GAL4/UAS-Neph1_V5, pros-GAL4/UAS-Neph2_V5 and pros-GAL4/UAS-Neph3_V5. N = 3. (TIF) [file pone.0040300.s001.tif]

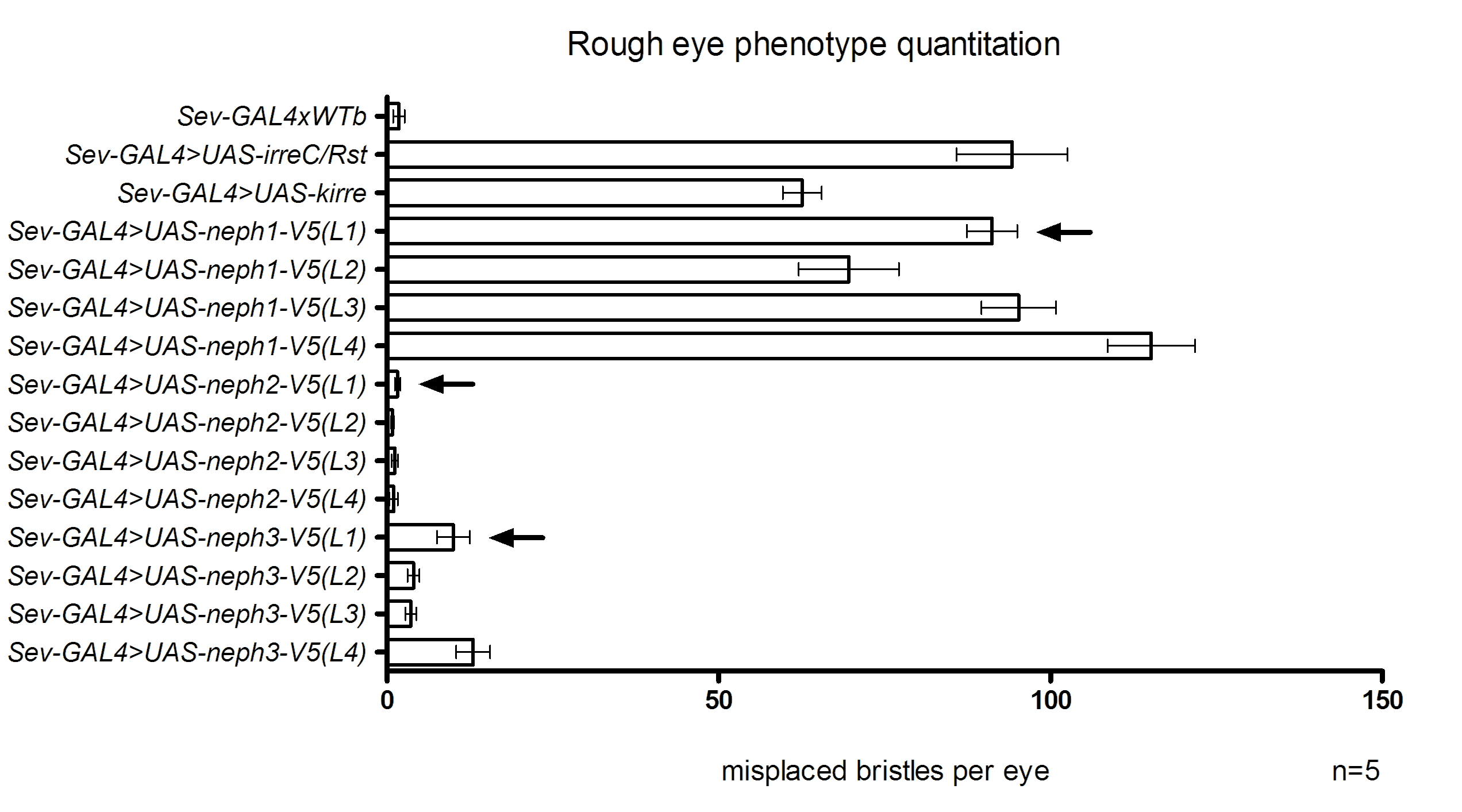

Supplement: Figure S2 — Rough eye phenotype quantitation. To quantify the rough eye phenotype, clusters of bristles were counted. The severity of the rough eye phenotype in flies misexpressing Neph1 is comparable to that in flies overexpressing IrreC/Rst. For each Neph protein data of four independent insertions are shown. Arrows are marking the lines selected for the rescue experiments and qRT-PCR. (TIF) [file pone.0040300.s002.tif]

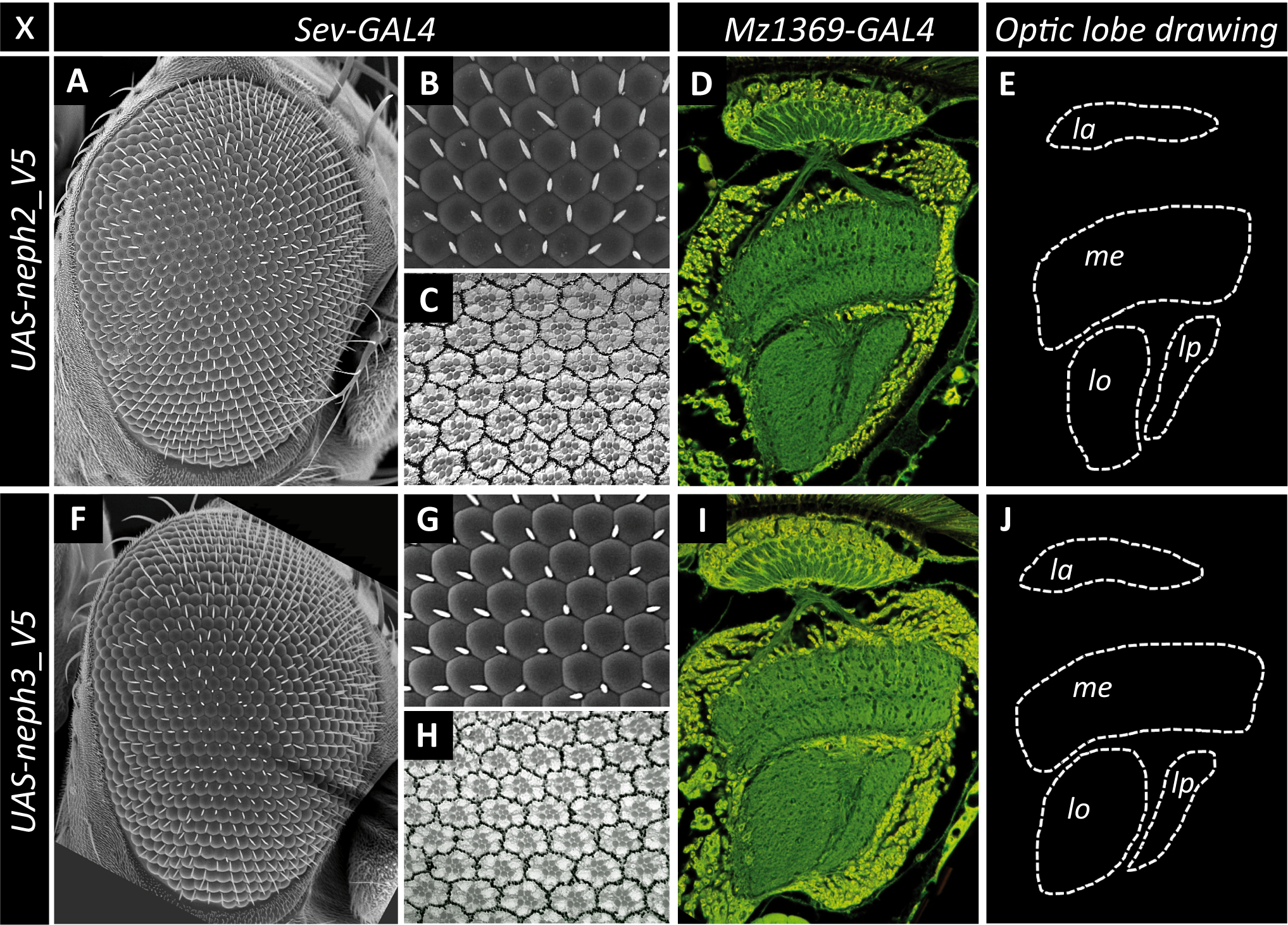

Supplement: Figure S3 — Neph2 and Neph3 cannot mimic neuronal and eye phenotypes of overexpressed Kirre or IrreC/Rst. Scanning electron micrographs of adult Drosophila eyes (A,B,F,G), close-up of the eye (B,G) and light micrographs of semithin sections (C,H). sev-GAL4 induced misexpression of Neph2 does not cause a rough eye phenotype (A,B). sev-GAL4 induced misexpression of Neph3 leads to a weak increase in the amount of misplaced bristles (F,G,H),(see Figure S2). Genotypes: sev-GAL4/UAS-neph2_V5 (A,B,C), sev-GAL4/UAS-neph3_V5 (F,G,H). Auto fluorescence micrographs of adult Drosophila optic lobes (D,I). The optic lobes show the typical wildtype-like arrangement of the neuropils. Optic lobe drawing: la: lamina, me: medulla, lo: lobula, lp: lobula plate. Genotypes: Mz1369-GAL4/UAS-neph2_V5 (D), Mz1369-GAL4/UAS-neph3_V5 (I). (TIF) [file pone.0040300.s003.tif]
